# Immediate and sustained root caries prevention of fluoride varnish combined with toothpastes: findings of clinical relevance?

**DOI:** 10.1038/s41432-024-01023-5

**Published:** 2024-05-31

**Authors:** Patrick Quinn, Brett Duane

**Affiliations:** School of Dental Science, Trinity College Dublin, Dublin, Ireland

**Keywords:** Dental caries, Fluoride varnish

## Abstract

**Design:**

An in vitro study to determine the immediate and sustained effect of fluoride varnish and its combination with fluoride toothpastes in preventing the development of root caries.

**Case selection:**

Human root dentine samples (150) were randomly divided into five experimental protocols of 30 specimens each: 1) fluoride varnish (22,600 ppm fluoride and 1–5% CPP-ACP); 2) fluoride varnish followed by Paste One (1100 ppm sodium fluoride and CPP-ACP); 3) fluoride varnish followed by Paste Plus (900 ppm sodium fluoride and CPP-ACP); 4) fluoride varnish followed by Paste One and Paste Plus; and 5) no treatment (control). A layer of varnish was applied to specimens except the control group and was left in situ for 18 h. The varnish layer was removed, and the various toothpaste treatments were initiated. Half of the specimens in each group were assigned to a short-term incubation model in which they were immediately subjected to a 7-day cariogenic challenge consisting of a combination of human saliva and artificial saliva containing 2% sucrose. The other half of the specimens in each group were assigned to the long-term incubation model in which the experimental protocol was continued for 8 weeks before initiating the seven-day cariogenic challenge. The protocols were evaluated by assessing dentine porosity (rhodamine intensity), mineral density, biofilm biomass, and viability assays.

**Data analysis:**

Confocal laser scanning microscopy was used to determine dentine porosity and Levene’s test was used to verify the assumption of equality of variances and normal distribution of errors before two-way ANOVA and the Games-Howell test were carried out at a significance level of 0.05 for both incubation models. Microcomputed tomography was used to determine mineral density with statistical analysis involving Levene’s test, two-way ANOVA and Tukey’s test at a significance level of 0.05 for both incubation models. Biomass was evaluated using a biofilm biomass assay with analysis of optical density data using Levene’s test, ANOVA and Scheffe’s test at a significance level of 0.05.

**Results:**

For both the short- and long-term incubation models, all the experimental regimes resulted in a statistically significant decrease in dentine porosity and an increase in mineral density when compared to the control group. Fluoride varnish followed by both pastes and fluoride varnish followed by Paste One resulted in a statistically significant decrease in dentine porosity for some depths in both models when compared to fluoride varnish alone. Changes in dentine porosity and mineral density were observed within groups over time. All the experimental regimes demonstrated anti-biofilm effects. Immediate and sustained anti-caries effects were observed for all preventive protocols, with the combination of fluoride varnish and Paste One resulting in superior additional anti-caries effects.

**Conclusions:**

The authors concluded that all protocols demonstrated immediate and sustained anti-caries effects against the development of root caries despite variations in effects over time. The combination of fluoride varnish and Paste One resulted in additional anti-caries effects that were consistently superior, with no additional effects being observed when Paste Plus was added in combination. The authors suggest that, within the study’s limitations, topical fluoride varnish seems to have a protective effect on root surfaces for up to eight weeks and that fluoride varnish should be considered as an important adjunct strategy in the prevention of root caries in older adults.

## A Commentary on

**Zamperini C A, Bedran-Russo A K**.

Immediate and sustained root caries prevention of fluoride varnish combined with toothpastes. *Caries Res* 2023. 10.1159/000533279

**GRADE Rating**: 

## Commentary

Effective root caries prevention strategies are becoming increasingly important as an estimated one-third of the global elderly population is now affected by root caries with the possibility of experiencing pain, tooth loss, and impacts on quality of life^1^. A recent systematic review and network meta-analysis by Zhang et al. summarised the direct and indirect clinical evidence on the effectiveness of both professionally applied and self-applied topical fluorides in the prevention of root caries^1^. The authors included 9 clinical trials involving 4030 participants and, when compared to controls, 38% silver diamine fluoride solution, 5% sodium fluoride varnish, and 1.2% acidulated phosphate fluoride were found to reduce root caries increment after 2 years. The use of fluoride mouth rinse and fluoride toothpaste, either alone or in combination, reduced root caries increment after one year. In terms of professionally applied topical fluorides, the authors found that an annual application of 38% silver diamine fluoride in combination with oral health education was most likely to be most effective in preventing root caries. Among the self-applied topical fluoride methods, the daily use of 0.2% sodium fluoride mouth rinse was most likely to be the most effective.

In vitro studies such as that conducted by Zamperini and Bedran-Russo^2^ are important early stages in the development of new preventive strategies to augment the existing body of evidence in this area and it is important that they are conducted to the highest standards so that subsequent clinical studies are as unbiased as possible^3^. However, to date there has been little guidance regarding the appraisal of in vitro studies^3^. Faggion has proposed a 14-item checklist that is a modification of the CONSORT checklist and this provides a useful structure for evaluating the in vitro study that is the subject of this commentary^3^.

The authors provide a very clear and comprehensive abstract, and the introduction sets out the background to the study as well as the three null hypotheses: 1) fluoride varnish will not exhibit immediate and sustained protective effects against the development of root caries; 2) root caries progression will not be further inhibited by the different combinations of fluoride varnish with toothpastes; and 3) the protective effects of the different combinations of varnish with toothpastes will not change over time. In terms of methods, the various interventions are described in detail, outcomes are clear, and comprehensive information is provided on the statistical tests that were used. However, the authors did not state why they chose a sample size of 150 root dentine specimens, so it is not possible to tell if the study was adequately powered to detect true differences. This is important as sample size can affect the precision of results, with smaller studies tending to result in wider confidence intervals and less precise results^3^. The authors also refer to randomly dividing the samples into the various experimental protocols, but fail to provide details regarding sequence generation, methods to conceal the sequence, the division of roles in terms of implementing the sequence, and any blinding that may have been utilised. Therefore, it is difficult to tell the extent to which measures were taken to minimise bias at this important stage in a study that relies on random allocation in terms of its design. Another reporting limitation is that confidence intervals are not clearly stated when reporting key findings but are limited to diagrammatic representation in the graphs that are provided. However, there is a clear statement regarding funding, and while no information is given regarding accessing the study protocol, the corresponding author provides contact details for further inquiries.

Zamperini and Bedran-Russo acknowledge a number of limitations of their study such as the inability of an in vitro model to mimic dynamic effects such as salivary clearance, the failure to simulate a high caries risk scenario as sugar was only renewed twice daily in the cariogenic challenge, issues relating to the overestimation of biomass associated with the biofilm model utilised, and the potential contribution of CPP-ACP to the results of the study as highlighted by previous research. It is also suggested by the present authors that a number of features of the study’s design may not accurately reflect the ‘real world’ environment. Firstly, after 18 h the fluoride varnish layer was removed using a surgical blade before implementation of the various protocols, but in a ‘real world’ scenario this would not take place as the fluoride varnish layer would likely reduce over time through normal oral activity and oral hygiene measures. Secondly, the long-term protocol involved implementing a seven-day cariogenic challenge after a period of eight weeks had elapsed, but this is unlikely to reflect reality as most people are likely to encounter cariogenic challenge on an ongoing daily basis and not experience a period of eight weeks free of challenge. Zamperini and Bedran-Russo suggest a sustained protective effect of fluoride varnish on root surfaces for up to eight weeks after application, but it would be interesting to see what impact, if any, sustained cariogenic challenge for the eight weeks would have on the results of the study.

In conclusion, this study contributes to an increasingly important area of dental research and the authors do not overstate their findings by stating that the application of fluoride varnish seems to be an important adjunct strategy in the prevention of root caries. However, the study has a number of limitations, and it is suggested that further in vitro research more closely simulating the ‘real world’ environment followed by clinical studies such as randomised controlled trials would be required before definitive recommendations can be made.

Practice points

- This in vitro study suggests that using fluoride toothpastes as well as fluoride varnish may have a role as an adjunct strategy in the prevention of root caries rather than just fluoride varnish alone, but the study has a number of limitations and further in vitro and clinical research would be required before making definitive recommendations.
- In vitro studies play a key role in informing subsequent clinical research such as randomised controlled trials and it is therefore important that they are reported to as high a standard as possible.
